# The Role of Airways 17β-Estradiol as a Biomarker of Severity in Postmenopausal Asthma: A Pilot Study

**DOI:** 10.3390/jcm9072037

**Published:** 2020-06-29

**Authors:** Giulia Scioscia, Giovanna Elisiana Carpagnano, Donato Lacedonia, Piera Soccio, Carla Maria Irene Quarato, Luigia Trabace, Paolo Fuso, Maria Pia Foschino Barbaro

**Affiliations:** 1 Department of Medical and Surgical Sciences, University of Foggia, 71122 Foggia, Italy; giulia.scioscia@unifg.it (G.S.); piera.soccio@unifg.it (P.S.); carlamariairene.quarato@gmail.com (C.M.I.Q.); paolo.fuso91@gmail.com (P.F.); mariapia.foschino@unifg.it (M.P.F.B.); 2 Institute of Respiratory Diseases, Policlinico Riuniti of Foggia, 71122 Foggia, Italy; 3 Department of Basic Medical Sciences, Neuroscience and Sense Organs, Section of Respiratory Disease, University “Aldo Moro” of Bari, 70121 Bari, Italy; elisiana.carpagnano@uniba.it; 4 Department of Clinical and Sperimental Medicine, University of Foggia, 71122 Foggia, Italy; luigia.trabace@unifg.it

**Keywords:** estradiol, severe asthma, postmenopausal asthma, sputum

## Abstract

Background: Asthma severity differs according to gender; in adult women, there is higher prevalence and severity of asthma than in men, and it coincides with changes in sex hormones. Recently, a new phonotype of asthma has been identified that appears after menopause, and it may be associated with decreased estrogen levels. Our goal was to study the 17β-estradiol (E2) concentrations in the blood and airways of women affected by asthma onset after menopause, evaluating its possible role in the severity of the disease. Methods: We enrolled 33 consecutive women with a diagnosis of postmenopausal asthma, recruited from the outpatient pulmonary clinic: 18 with severe (SA) and 15 with mild-to-moderate (MMA) asthma. We also included 30 age-matched healthy menopausal women as controls (HS). All subjects enrolled underwent blood and sputum collection (IS), and E2 concentrations were determined in plasma and sputum supernatant samples using an enzyme-linked immunosorbent assay (ELISA) kit. Results: Significantly higher serum concentrations of E2 were found in postmenopausal SA compared to MMA and HS, respectively (33 ± 5.5 vs. 24 ± 6.63 vs. 7.79 ± 1.54 pg/mL, *p* < 0.05). Similar results were found in the IS: significantly higher levels of E2 were detected in patients with postmenopausal SA compared with MMA and HS, respectively (0.34 ± 0.17 vs. 0.26 ± 0.13 vs. 0.07 ± 0.06 pg/mL, *p* < 0.05). We found positive correlations between IS E2 concentrations and sputum neutrophil levels in SA group (*ρ* = 0.52, *p* < 0.05). Conclusions: Our findings showed the possibility to measure E2 in the airways, and it has increased in postmenopausal asthmatic patients, especially in those with SA. Airways E2 levels may serve as a suitable biomarker of postmenopausal SA to help to phenotype SA patients with neutrophil inflammation.

## 1. Introduction

Gender differences in the incidence, prevalence and severity of asthma are reported by epidemiological data. These differences also change throughout life. During childhood, the prevalence of asthma shows a higher risk in boys compared to girls. Otherwise, after puberty, there is an increase of asthma incidence in women until menopause [1,2]. The causes of gender differences are unknown; however, the hypothesis of the possible link between the higher incidence of asthma in women and changes in sex hormones was evaluated.

Three types of estrogen molecules are produced in human females: estradiol, estrone and estriol. Estradiol is the major female sex hormone, and its levels are predominant during a woman’s reproductive years. Conversely, during the menopausal period, the amount of estradiol declines to very low levels. Moreover, in pregnant women, there is a large amount of estriol, while estrone becomes predominant in menopause.

Recent studies proved that estrogen increases the T helper (Th) 2 response in the presence of allergens, likely by stimulating the proliferation and differentiation of Th cells into Th2 cells [3].

Another mechanism is that the presence of physiologic concentrations of 17β-estradiol seem to induce mast cells’ degranulation and to enhance allergen crosslinking with surface IgE [4,5]. On the other hand, testosterone has been shown to attenuate type 2 innate lymphoid cells’ (ILC2) function and proliferation [6].

Estradiol may increase the release of IL-1β and TNF-α and, at the same time, decrease the release of IL-10, promoting inflammation [7]. Instead, progesterone may cause eosinophilia, as it significantly increases the IL-10, IL1-β and TNF-α amounts in the lungs and the release of IL-4 by bone marrow cells [8].

Women with severe asthma also showed a higher IL-23R expression and IL-17A production by Th17-differentiated cells when compared with severely asthmatic men [9]. A similar molecular environment was reproduced in ovariectomized mice receiving 17β-estradiol [9].

A new phenotype of asthma with onset after menopause has been recently described for a subset of women [10,11], but the mechanisms that initiate and regulate postmenopausal asthma remain largely unknown. Among women over 50 years of age, menopause can either coincide with the onset of asthma or be associated with the deterioration of a preexisting asthma condition [11]. After menopause, estrogen levels decrease to the levels observed in patients with surgical oophorectomy, who also show extremely low progesterone levels. The incidence of asthma may be associated with the abrupt decrease in estrogen levels and the consequent impairment in the hypothalamic-pituitary-gonadal axis that occurs during the menopausal transition.

Menopausal-onset asthma affects 18% of the total female asthma population, and frequently, it is characterized by the absence of atopy; recurrent sinusitis; aspirin sensitivity and/or intolerance to angiotensin-converting enzyme inhibitors, greater severity (use of systemic steroids to control symptoms and frequent hospitalizations) and an altered perception of asthmatic symptoms [11,12].

All sex steroid hormone receptors were found in the lungs: estrogen receptor (ER-α or ER-β), progesterone receptor (PR-A or PR-B) and an androgen receptor (AR). The activation of steroid hormones occurs as a result of the link with their own unique receptors. Among different estrogens, estradiol is the one having the greatest affinity for estrogen receptors and the same binding affinity for both ER-α and ER-β [8].

Sex hormone-binding globulin (SHBG) is an important steroid hormone binding protein in human plasma and regulates sex hormone delivery to tissues and cells by binding them and keeping them inactive.

Under normal conditions, in the blood stream, testosterone (T) and 17β-estradiol (E2) are largely bound to SHBG. Instead, a smaller part (~2%) is dissociated from SHBG, and the nonbound hormone is the biologically active fraction [13].

The aim of our study was to analyze the blood and airways’ E2 concentrations of women affected by asthma onset after menopause, evaluating its possible role in the severity of the disease and comparing it with other inflammatory biomarkers.

## 2. Methods

### 2.1. Patients

Thirty-three consecutive women with postmenopausal asthma, 18 with severe and 15 with mild-to-moderate asthma, were recruited in this study from the outpatient facility of the Institute of Respiratory Disease of the University of Foggia, Italy. We also enrolled 30 age-matched healthy menopausal women as controls. Written informed consent was obtained from all subjects, and the study was approved by the institutional ethics committee of the University of Foggia (institutional review board approval No. 17/CE/2014).

All women were classified as postmenopausal on the basis of a completed questionnaire to confirm whether or not they had entered menopause (cessation of menstruation for 12 months). They were considered to be postmenopausal asthmatics if their asthma had started at menopause, i.e., in the period between 1 year before and 1 year after their last menstrual period. All patients with asthma were assessed within a period of stability and at least 4 weeks after an upper respiratory tract infection and were classified and treated according to Global Initiative for Asthma (GINA) guidelines [14]. Smokers, women on postmenopausal hormone replacement therapy (HRT) and asthmatics randomized in a controlled trial and/or with biologic therapy were excluded from the study.

At the first visit, a complete baseline questionnaire requesting information on medical history, menopausal status, hormone use and other reproductive and lifestyle variables was administered to all subjects. Subsequently, they received a physical examination, including body mass index (BMI) measurement, atopy assessment, exhaled nitric oxide (FENO) measurement and spirometry with a bronchial obstruction reversibility test. During the second visit, subjects underwent blood analysis for serum levels of E2 and cells count and, finally, sputum induction (IS). Hormones were dosed after a standardized rest, at the same time of day for all subjects, as they are known to be extremely dependent on circadian rhythm, stress, treatments, etc.

### 2.2. Menopausal Assessment

The menopausal status was assessed by a standardized self-administered questionnaire. All the participants were asked to indicate their putative mean menopause age and whether it was natural or surgical. In the case of physiological menopause, they were asked to enlist the date of their last period; otherwise, they were asked to indicate type, reason and date of surgical intervention. The women were also inquired about their medical history, with specific questions investigating about any concomitant state and relative treatment of conditions that may mimic menopausal symptoms, including depression, anemia, hypothyroidism, diabetes, previous contraceptives uses and concurrent hormone replacement therapy (HRT). As an exclusion criterion, women in concurrent HRT were not enrolled in the study. The last item of the questionnaire concerned the onset of asthma symptoms, also investigating if any respiratory symptoms had ever been previously experimented in conjunction with the menstrual cycle or eventual pregnancies. In the case of doubts about a preexisting asthma worsened at menopause, patients were excluded from the study too.

### 2.3. Atopic Status

All the enrolled subjects were skin prick-tested, and the atopic status was assessed as SPOT positiveness. Skin prick tests (SPTs) were performed using a standard panel for common aeroallergens (Lofarma, Italy) [15].

### 2.4. Lung Function

Pulmonary function tests were performed. Forced expiratory volume in one second (FEV1), forced vital capacity (FVC) and plethysmographic lung volumes were measured using a spirometer (Sensormedics, Milan, Italy) following international standards in all subjects [16,17]. The best value of three maneuvers was expressed as a percentage of the predicted normal value. After the baseline evaluation, spirometry was repeated 15 min after the subjects had inhaled 400 mg of salbutamol, as previously reported. The reversibility of airways obstruction was expressed in terms of the percent changes from the baseline of FEV1.

### 2.5. Measurement of FENO

The Medisoft FENO+ device (Medisoft Belgium, Sorinnes, Belgium), which is a semiportable for repeatable multiflow measurements of exhaled NO with offline measurements, was used. It has a software package that provides step-by-step online quality control. The measurement range is 0–600 ppb. FENO was measured using a previously described restricted breath technique, which employed expiratory resistance and positive mouth pressure to close the velum and exclude nasal NO; expiratory flow measurements at 50 mL/s and a 350 mL/s have been evaluated. Repeated exhalations were performed until three plateaus agreed within 5% [18].

### 2.6. IS Collection and Processing

According to the method described by Toungoussova et al. [19], sputum was induced through the inhalation of hypertonic saline solution (4.5%) with an ultrasonic nebulizer (DeVilbiss Healthcare GmbH, Mannheim, Germany) and analyzed after the selection of mucus plugs. In patients with severe asthma, we used spontaneous sputum when they were particularly uncontrolled. Ten healthy subjects, 9 patients with severe asthma and 5 with mild-to-moderate asthma, were not able to produce adequate sputum samples (defined as containing at least 500 non-squamous cells), and their samples were discarded. The sputum (spontaneous or induced) was used for cytologic analysis, and the supernatant was used for E2 analysis.

### 2.7. 17-β-estradiol (E2) Analysis

A venous blood sample was drawn from each participant between 8 a.m. and 10 a.m., and the serum was obtained by centrifugation. Serum aliquots were then stored at −80 °C until analysis. The E2 concentration was determined in plasma and sputum supernatant samples using an enzyme-linked immunosorbent assay kit (Estradiol Serum EIA KIT, catalog number KB30-H1, Arbor Assays, Ann Arbor, MI, USA) as described in the manufacturer’s instructions. Intra and interassay coefficients of variation were 4.1% and 9.6%, respectively.

### 2.8. Statistical Analysis

Descriptive statistics (i.e., means, standard deviations and percentages) were applied to summarize the continuous and categorical variables. One-way ANOVA and Kruskal–Wallis rank tests were used to compare groups. Correlation between variables was measured using the Spearman rank correlation test. Significance was defined as a *p*-value of <0.05. SPSS 22.0 (III INC, Chicago, IL, USA) was used to store and analyze the data.

## 3. Results

Anthropometric, clinical, functional and biologic data of subjects enrolled are reported in Table 1.

**Figure 1:**
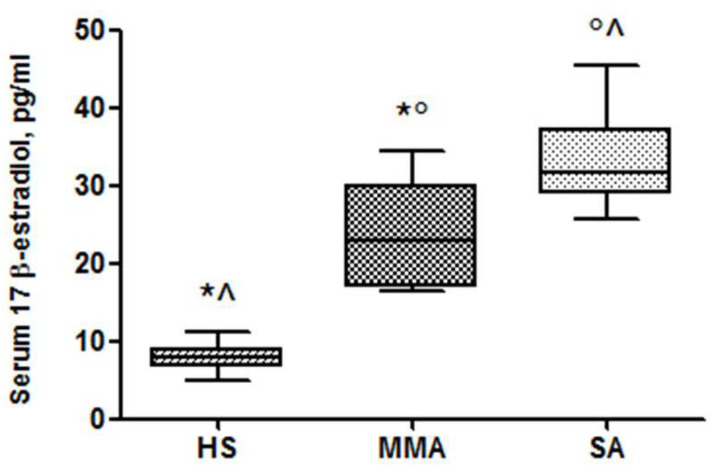
Serum concentrations of 17β-estradiol in healthy subjects (HS), mild-to-moderate asthma (MMA) and severe asthma (SA) groups. * *p* < 0.05 HS vs. MMA, ^ *p* < 0.05 HS vs. SA and ° *p* < 0.05 MMA vs. SA.

Higher serum concentrations of E2 were found in postmenopausal SA compared to MMA ones and healthy women (33 ± 5.5 vs. 24 ± 6.63 vs. 7.79 ± 1.54 pg/mL, *p* < 0.05) (Figure 1).

**Figure 2:**
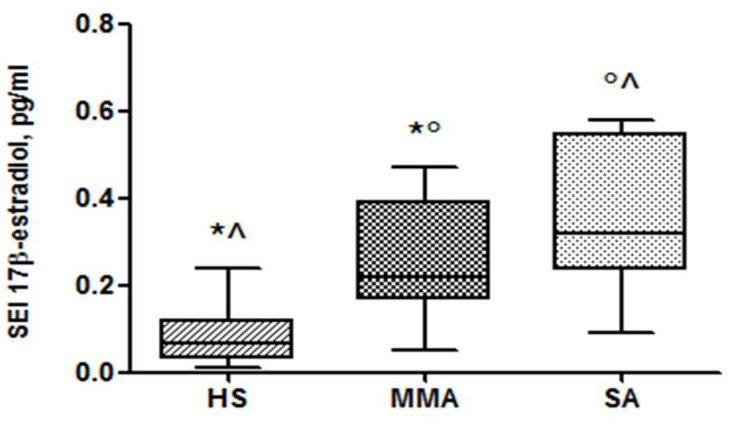
Induced sputum supernatant (SEI) concentrations of 17β-estradiol in HS, MMA and SA groups. * *p* < 0.05 HS vs. MMA, ^ *p* < 0.05 HS vs. SA and ° *p* < 0.05 MMA vs. SA.

We found that E2 was detectable in all IS supernatant samples collected: 9 patients with SA, 10 patients with MMA and 20 HS. Similar results were found in the IS; i.e., significantly higher levels of E2 were found in patients with postmenopausal SA compared to MMA and HS, respectively (0.34 ± 0.17 vs. 0.26 ± 0.13 vs. 0.07 ± 0.06 pg/mL, *p* < 0.05) (Figure 2).

In patients with postmenopausal SA, we found a positive correlation between IS E2 and neutrophil IS levels (*ρ* = 0.52, *p* < 0.05).

We did not find a significant correlation between serum and IS E2 levels and any correlations between serum and IS E2 with other clinical variables, such as BMI, ACT and ACQ scores, FEV1 and FENO.

## 4. Discussion

The main findings of this study were (1) the identification of higher serum concentrations of E2 in postmenopausal SA patients than in MMA and healthy menopausal ones, (2) for the first time, to our knowledge, the detection of E2 free levels in IS supernatants with higher concentrations in SA patients than MMA and HS, like in the serum samples and (3) the presence of a positive correlation of IS levels of E2 with IS neutrophils counted in the SA group.

Several studies aimed to understand the role of sex hormones in lung inflammatory processes [20]. Asthma is a chronic inflammatory disease characterized by a sexual dimorphism. In older adults, two phenotypes are usually observed: patients with long-standing asthma who develop additional airflow limitations and those who develop late-onset asthma [21].

During the menopausal period, the decline in sex hormones synthesis causes a loss of the hypothalamic feedback inhibition and a marked increase in gonadotropin-releasing hormone (GnRH) levels. However, data from a research study showed lower serum FSH and LH levels in menopausal women with new-onset asthma or preexisting lung disease compared to nonasthmatic postmenopausal women [22]. Furthermore, patients with secretion dysfunctions of cortical hormones have shown a more severe asthmatic phenotype characterized by repeated asthma attacks; these patients become more dependent on exogenous glucocorticoids [23].

These findings could justify the higher serum levels of E2 in postmenopausal SA patients than in MMA and healthy menopausal ones.

Under normal conditions, in the blood stream, testosterone (T) and 17β-estradiol (E2) are largely bound to SHBG. Instead, a smaller part (~2%) is dissociate from SHBG [13].

The 20–40% of circulating E2 is bound to SHBG that controls the sex hormone transport to tissues and cells. According to the free hormone hypothesis, the unbound quota is biologically active and freely diffuses across the cell surface membranes [24]. For the first time, in our pilot study, we detected E2 free levels in the IS supernatants, and the preliminary results confirm that menopausal SA shows higher concentrations than MMA and healthy menopausal subjects, regardless of the sputum collection method (spontaneous or induced). Indeed, the difference between the spontaneous or induced sputum is only in the collection’s method, the latter being conducted by the help of the inhalation of a nebulized sterile saline solution (isotonic or hypertonic). Sputum cellular compositions and protein marker detections are not influenced by the method of collection, as previously reported by other studies in the literature [25].

We did not find a correlation between the serum and IS samples, probably due to the small number of patients.

Studies have shown that sex hormones can regulate airway inflammations, and estrogen increased Th2-mediated airway inflammations, although these effects are still under investigation.

Type 2 inflammation in asthma occurs in many patients but not all. In some patients with asthma is found an increase of IL-17A that seems associated with more severe forms of asthma [26]. CD4+ T helper, 17 (Th17) cells, γδ T cells, neutrophils and innate lymphoid cells are able to produce IL-17A in the lungs.

Newcomb DC et al. [9] found an increased production of IL-17A from Th17 cells in severe asthmatics women compared to men. Moreover, using a mouse model, the same authors showed that a rise of IL-17A is associated with an increased neutrophilic airway inflammation in female mice compared to male mice when stimulated by E2.

Consistent with the literature, we demonstrated a positive correlation between IS E2 and neutrophil IS levels in patients with postmenopausal SA, and our results confirm that neutrophils are considered to be central to the pathogenesis of most forms of severe late-onset asthma.

Nevertheless, more studies are needed to better understand the role of sex hormones in IL-17A-mediated airway inflammation and neutrophilic asthma.

Estrogens are shown to possess the ability to inhibit the chemotactic activity of polymorphonuclear leukocytes; on the contrary, progesterone increases it [27]. Furthermore, progesterone and testosterone may reduce the ROS and NO generation driven by estradiol [28,29]. Therefore, increased levels of E2 in the respiratory tract might fix neutrophils to the lower airway epithelium and give rise to local oxidative stress.

Additionally, in studies on human leukocytes, it has been shown that estrogens reduce the process of programmed cell death (i.e., apoptosis), while testosterone seems to favor it. This could allow the survival of self-reactive cells and, at least in part, explain the greater predisposition of women for Th17 and Th1-driven autoimmune diseases, such as rheumatoid arthritis (RA) and systemic lupus erythematosus (SLE) [30].

Gender-specific asthma pathogenesis may also be confounded by other comorbidities. The Severe Asthma Research Program (SARP) and the European Network for Understanding Mechanisms of Severe Asthma showed a higher body mass index (BMI) and neutrophilic inflammation among women with severe asthma compared to nonsevere asthma but not in men [31,32]. Additionally, in our study, female patients with severe asthma showed a slightly higher, if not statistically significant, BMI compared to those with mild-moderate asthma. Additionally, whether or not gender dimorphism is related to female hormones remains unclear. One possible explanation for the obese-asthma neutrophilic phenotype may be related to serum levels of leptin, a key player in body weight regulation, which increases helper T cell type 1 (Th1) inflammation [33]. However, several studies have shown a strong correlation between BMI and nonatopic asthma only in women of childbearing age and not in men. On the contrary, no gender difference has been reported in the incidence of atopic asthma [34,35]. Taken together, these data suggest the possibility of an interaction between gender, age, BMI and asthma type (atopic vs. nonatopic) in the pathogenesis of severe asthma in females.

The strengths of our preliminary study are the detection of E2 free levels in the IS supernatants of asthmatic patients and healthy controls and the study of clinical and inflammatory characteristics of a well-described phenotype that is postmenopausal asthma.

However, the study also has key limitations, such as the small number of subjects enrolled and the absence of a validation of E2 free levels detection technique in the sputum.

## 5. Conclusions

In conclusion, this preliminary study suggests that E2 plays a role in postmenopausal severe asthma. This hormone is detectable directly in the airways, and it may be a noninvasive marker to phenotype SA patients not eligible for actual biologic treatments with neutrophilic inflammation.

## Figures and Tables

**Table 1:** Baseline characteristics of the study population.

Number of Patients	HS 30	MMA 15	SA 18
Demographic and clinical characteristics			
Age, years (mean ± SD)	62 ± 5.45	55 ± 18	58 ± 11
BMI, Kg/m^2^, (mean ± SD)	27 ± 5	26 ± 5	28 ± 5
Age of onset, years (mean ± SD)		45 ± 8	40 ± 15
Postmenopausal status (%)	25 (83%)	12 (80%)	14 (78%)
Surgical menopause (%)	5 (17%)	3 (20%)	4 (22%)
Exacerbations/year, (mean ± SD)		1 ± 1 *	3 ± 1 *
Atopy (SPT+), *n* (%)	0 (0%)	5 (33.3%)	7 (38.8%)
Aspirin-sensitivity, *n* (%)		8 (53.3%)	10 (55.5%)
ICS low to medium dose, *n* (%)		12 (80%)	0
ICS high dose/LABA, *n* (%)		3 (20%) *	7 (38.8%) *
ICS high dose/LABA/TIOTROPIUM, *n* (%)		0	11 (61.1%)
OCS, *n* (%)		0	10 (55.5%)
ACT		19 ± 3 *	14 ± 4 *
ACQ		1 ± 0.7 *	3 ± 2 *
Lung function			
FEV1 preBD, % predicted	85 ± 5 ^†,¥^	78 ± 14 *^,¥^	67 ± 19 ^†,^*
FEV_1_/FVC preBD, %	82 ± 12 ^†,¥^	68 ± 10 ^¥^	62 ± 12 ^†^
Reversibility, %	5 ± 4.5 ^†,¥^	13 ± 9 ^¥^	11 ± 6 ^†^
TLC, % predicted	104 ± 13	98 ± 14	96 ± 14
RV, % predicted	89 ± 20	97 ± 25	110 ± 26
Biomarkers			
FENO_50_, ppb	15 ± 6 ^†,¥^	22 ± 28 ^¥^	25 ± 21^†^
Blood eosinophil count, cells/mL	0.05 ± 0.22 ^†,¥^	0.21 ± 0.24 ^¥^	0.25 ± 0.25 ^†^
Blood neutrophil level, cells/mL	2.2 ± 1.7 ^†^	2.7 ± 1.4 *	5.6 ± 2.7 ^†,^*
Eosinophil IS count, % total cells	1 ± 1 ^†,¥^	2 ± 2 ^¥^	3 ± 2 ^†^
Neutrophil IS level, % total cells	38 ± 18 ^†^	37 ± 12 *	68 ± 15 ^†,^*
Serum 17β-estradiol, pg/mL	7.79 ± 1.54 ^†,¥^	24 ± 6.63 *^,¥^	33 ± 5.5 ^†,^*
SEI 17β-estradiol, pg/mL	0.07 ± 0.06 ^†,¥^	0.26 ± 0.13 *^,¥^	0.34 ± 0.17 ^†,^*

Abbreviations. HS: healthy subjects, MMA: mild-moderate asthma, SA: severe asthma, BMI: body mass index, SPT+: SPOT positiveness, ICS: inhaled corticosteroids, ICS/LABA: inhaled corticosteroids/long-acting beta-adrenoceptor agonists, OCS: Oral Corticosteroids, ACT: asthma control test, ACQ: asthma control questionnaire, FEV1: forced expiratory volume, FEV1/FVC: forced expiratory volume/forced vital capacity, BD: Bronchodilator, FEF: forced expiratory flow, Raw: airway resistance, TLC: total lung capacity, RV: residual volume, RV/TLC: residual volume/total lung capacity, IS: induced sputum and SEI: induced sputum supernatant. ^†^ *p* < 0.05 between HS and SA, * *p* < 0.05 between SA and MMA and ^¥^ *p* < 0.05 between HS and MMA.

## References

[1] Fuseini H., Newcomb D.C. (2017). Mechanisms Driving Gender Differences in Asthma. Curr. Allergy Asthma Rep..

[2] Zein J.G., Denson J.L., Wechsler M.E. (2019). Asthma over the Adult Life Course: Gender and Hormonal Influences. Clin. Chest Med..

[3] Uemura Y., Liu T.Y., Narita Y., Suzuki M., Matsushita S. (2008). 17β-Estradiol (E2) plus tumor necrosis factor-α induces a distorted maturation of human monocyte-derived dendritic cells and promotes their capacity to initiate T-helper 2 responses. Hum. Immunol..

[4] Zaitsu M., Narita S.I., Lambert K.C., Grady J.J., Estes D.M., Curran E.M., Brooks E.G., Watson C.S., Goldblum R.M., Midoro-Horiuti T. (2007). Estradiol activates mast cells via a non-genomic estrogen receptor-α and calcium influx. Mol. Immunol..

[5] Bonds R.S., Midoro-Horiuti T. (2013). Estrogen effects in allergy and asthma. Curr. Opin. Allergy Clin. Immunol..

[6] Cephus J.Y., Stier M.T., Fuseini H., Yung J.A., Toki S., Bloodworth M.H., Zhou W., Goleniewska K., Zhang J., Garon S.L. (2017). Testosterone Attenuates Group 2 Innate Lymphoid Cell-Mediated Airway Inflammation. Cell Rep..

[7] de Oliveira A.P., Domingos H.V., Cavriani G., Damazo A.S., dos Santos Franco A.L., Oliani S.M., Oliveira-Filho R.M., Vargaftig B.B., de Lima W.T. (2007). Cellular recruitment and cytokine generation in a rat model of allergic lung inflammation are differentially modulated by progesterone and estradiol. Am. J. Physiol. Cell Physiol..

[8] Tam A., Morrish D., Wadsworth S., Dorscheid D., Man S.P., Sin D.D. (2011). The role of female hormones on lung function in chronic lung diseases. BMC Womens Health.

[9] Newcomb D.C., Cephus J.Y., Boswell M.G., Fahrenholz J.M., Langley E.W., Feldman A.S., Zhou W., Dulek D.E., Goleniewska K., Woodward K.B. (2015). Estrogen and progesterone decrease let-7f microRNA expression and increase IL-23/IL-23 receptor signaling and IL-17A production in patients with severe asthma. J. Allergy Clin. Immunol..

[10] Balzano G., Fuschillo S., De Angelis E., Gaudiosi C., Mancini A., Caputi M. (2007). Persistent airway inflammation and high exacerbation rate in asthma that starts at menopause. Monaldi Arch. Chest Dis = Arch. Monaldi per le Mal del Torace.

[11] Foschino Barbaro M.P., Costa V.R., Resta O., Prato R., Spanevello A., Palladino G.P., Martinelli D., Carpagnano G.E. (2010). Menopausal asthma: A new biological phenotype?. Allergy.

[12] Baldaçara R.P., Silva I. (2017). Association between asthma and female sex hormones. Sao Paulo Med. J..

[13] De Ronde W., Van Der Schouw Y.T., Muller M., Grobbee D.E., Gooren L.J., Pols H.A., De Jong F.H. (2005). Associations of Sex-Hormone-Binding Globulin (SHBG) with Non-SHBG-Bound Levels of Testosterone and Estradiol in Independently Living Men. J. Clin. Endocrinol. Metab..

[14] Global Strategy for Asthma Management and Prevention. www.ginasthma.org.

[15] Heinzerling L., Mari A., Bergmann K.C., Bresciani M., Burbach G., Darsow U., Durham S., Fokkens W., Gjomarkaj M., Haahtela T. (2013). The skin prick test—European standards. Clin. Transl. Allergy.

[16] Miller M.R., Hankinson J.A., Brusasco V., Burgos F., Casaburi R., Coates A., Crapo R., Enright P., Van Der Grinten C.P., Gustafsson P. (2005). Standardisation of spirometry. Eur. Respir. J..

[17] Wanger J., Clausen J.L., Coates A., Pedersen O.F., Brusasco V., Burgos F., Casaburi R., Crapo R., Enright P., Van Der Grinten C.P. (2005). Standardisation of the measurement of lung volumes. Eur. Respir. J..

[18] American Thoracic Society, European Respiratory Society (2005). ATS/ERS recommendations for standardized procedures for the online and offline measurement of exhaled lower respiratory nitric oxide and nasal nitric oxide, 2005. Am. J. Respir. Crit. Care Med..

[19] Toungoussova O., Migliori G.B., Barbaro M.P., Esposito L.M., Dragonieri S., Carpagnano G.E., Salerno F.G., Neri M., Spanevello A. (2007). Changes in sputum composition during 15 min of sputum induction in healthy subjects and patients with asthma and chronic obstructive pulmonary disease. Respir. Med..

[20] Assaggaf H., Felty Q. (2017). Gender, Estrogen, and Obliterative Lesions in the Lung. Int. J. Endocrinol..

[21] Fuentes N., Silveyra P. (2018). Endocrine regulation of lung disease and inflammation. Exp. Biol. Med. (Maywood).

[22] Della Torre F., Cassani L., Segale M. (1988). Asma ed orticaria nell’anziano: Ruolo degli ormoni ipofiso-gonadici in menopausa. Rass. Geriatr..

[23] Jiang Y., Zhou Z., Ji Y. (2014). Experimental immunology Effects of the recombinant allergen rDer f 2 on neuro-endocrino-immune network in asthmatic mice. Cent. Eur. J. Immunol..

[24] Mendel C.M. (1992). The free hormone hypothesis. Distinction from the free hormone transport hypothesis. J. Androl..

[25] Bhowmik A., Seemungal T.A.R., Sapsford R.J., Devalia J.L., Wedzicha J.A. (1998). Comparison of spontaneous and induced sputum for investigation of airway inflammation in chronic obstructive pulmonary disease. Thorax.

[26] Newcomb D.C., Peebles R.S. (2013). Th17-mediated inflammation in asthma. Curr. Opin. Immunol..

[27] Miyagi M., Aoyama H., Morishita M., Iwamoto Y. (1992). Effects of Sex Hormones on Chemotaxis of Human Peripheral Polymorphonuclear Leukocytes and Monocytes. J. Periodontol..

[28] Itagaki T., Shimizu I., Cheng X., Yuan Y., Oshio A., Tamaki K., Fukuno H., Honda H., Okamura Y., Ito S. (2005). Opposing effects of oestradiol and progesterone on intracellular pathways and activation processes in the oxidative stress induced activation of cultured rat hepatic stellate cells. Gut.

[29] Marin D.P., Bolin A.P., de Cassia Macedo dos Santos R., Curi R., Otton R. (2010). Testosterone suppresses oxidative stress in human neutrophils. Cell Biochem. Funct..

[30] Cutolo M., Sulli A., Capellino S., Villaggio B., Montagna P., Seriolo B., Straub R.H. (2004). Sex hormones influence on the immune system: Basic and clinical aspects in autoimmunity. Lupus.

[31] Abraham B., Antó J.M., Barreiro E., Bel E.H., Bonsignore G., Bousquet J., Castellsague J., Chanez P., Cibella F., Cuttitta G. (2003). The ENFUMOSA cross-sectional European multicentre study of the clinical phenotype of chronic severe asthma. Eur. Respir. J..

[32] Moore W.C., Meyers D.A., Wenzel S.E., Teague W.G., Li H., Li X., D’Agostino R., Castro M., Curran-Everett D., Fitzpatrick A.M. (2010). Identification of asthma phenotypes using cluster analysis in the severe asthma research program. Am. J. Respir. Crit. Care Med..

[33] Quek Y.W., Sun H.L., Ng Y.Y., Lee H.S., Yang S.F., Ku M.S., Lu K.H., Sheu J.N., Lue K.H. (2010). Associations of Serum Leptin with Atopic Asthma and Allergic Rhinitis in Children. Am. J. Rhinol. Allergy.

[34] Beuther D.A., Sutherland E.R. (2007). Overweight, obesity, and incident asthma: A meta-analysis of prospective epidemiologic studies. Am. J. Respir. Crit. Care Med..

[35] Ma J., Xiao L. (2013). Association of general and central obesity and atopic and nonatopic asthma in US adults. J. Asthma.

